# Enhancing Zn Deposition Reversibility on MXene Current Collectors by Forming ZnF_2_‐Containing Solid‐Electrolyte Interphase for Anode‐Free Zinc Metal Batteries

**DOI:** 10.1002/smll.202407226

**Published:** 2025-01-28

**Authors:** Chaofan Chen, Rui Guo, Swapna Ganapathy, Baukje Terpstra, Hao Wang, Zhibin Lei, Frans Ooms, Bart Boshuizen, Marnix Wagemaker, Lars J. Bannenberg, Xuehang Wang

**Affiliations:** ^1^ Department of Radiation Science and Technology Delft University of Technology Delft 2629 JB The Netherlands; ^2^ School of Materials Science and Engineering Shaanxi Normal University 620 West Chang'an Street Xi'an Shaanxi 710119 China; ^3^ Xi'an Rare Metal Materials Institute Co., Ltd Xi'an 710016 China

**Keywords:** anode‐free, electrolyte, MXene, solid electrolyte interphase, Zn deposition, Zn metal batteries

## Abstract

Anode‐free aqueous zinc metal batteries (AZMBs) offer significant potential for energy storage due to their low cost and environmental benefits. Ti_3_C_2_T*_x_* MXene provides several advantages over traditional metallic current collectors like Cu and Ti, including better Zn plating affinity, lightweight, and flexibility. However, self‐freestanding MXene current collectors in AZMBs remain underexplored, likely due to challenges with Zn deposition reversibility. This study investigates the combination of a Ti_3_C_2_T*_x_* self‐freestanding film with advanced electrolyte engineering, specifically examining the effects of Li‐salt and propylene carbonate (PC) as additives on Zn plating reversibility. While using Li^+^ ions as an additive alone facilitates uniform Zn deposition on bulk metals through the electrostatic shielding effect, the addition of Li‐salt negatively impacts Zn plating uniformity on Ti_3_C_2_T*_x_*. Meanwhile, using PC additive alone forms an organic SEI layer on Ti_3_C_2_T*_x_* and causes Zn agglomeration. The use of both additives together results in a ZnF_2_‐containing hybrid SEI layer with improved interfacial kinetics, promoting more uniform Zn deposition. This approach achieves an average Coulombic efficiency (CE) of 96.8% over 150 cycles (a maximum CE of 97.8%). The study highlights the strategic difference in electrolyte design, emphasizing the need for tailored approaches to optimize Zn deposition on MXenes, contrasting with traditional metallic current collectors.

## Graphical abstract

This study investigates the influence of electrolyte additives on Zn plating behavior on intercalative‐type 2D Ti_3_C_2_T_x_ MXene current collectors in anode‐free AZMBs. The co‐addition of Li‐salt and PC generates a ZnF_2_‐containing hybrid SEI layer that promotes homogeneous Zn deposition, achieving an average Coulombic efficiency of 96.8% for Zn plating/stripping over 150 cycles.

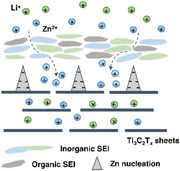

## 1 Introduction

The pursuit of advanced energy storage solutions with economic viability, environmental sustainability, and prolonged cycle life, has prompted extensive exploration of advanced electrochemical energy storage devices.^[^ ^1^, ^2^ ^]^ Moreover, concerns regarding the scarcity of lithium resources, escalating costs, and safety issues associated with organic electrolytes have intensified efforts to explore promising alternatives to current lithium‐ion batteries.^[^ ^3^, ^4^ ^]^ Aqueous zinc metal batteries (AZMBs) have emerged as promising devices for stationary energy storage due to their low cost, material abundance, and low toxicity.^[^ ^5^ ^]^ The relatively high theoretical capacity of 820 mAh g^−1^ (or 5855 mAh cm^−3^), the low redox potential of zinc metal (−0.762 V vs the standard hydrogen electrode), and good stability further enhance its appeal, positioning AZMBs as standout candidates for the next‐generation EES devices. Despite its high theoretical capacity and low redox potential, the practical energy densities of AZMBs are limited by the heavier battery designs due to the use of excessive Zn metal anodes (≥100 µm). Reasonable modifications on the Zn anode, including applying thinner Zn foil, pre‐depositing Zn on metallic or carbonaceous substrates, or especially exploring anode‐free configurations can reduce the weight and space demand for anode materials, improve the Zn utilization level, thereby enabling a higher energy density at potentially lower costs.^[^ ^6^ ^]^

Specifically, the anode‐free AZMBs are composed of an electron‐conductive current collector and a Zn‐rich cathode host. In this design, the traditional anode is replaced by a “Zn‐free” anode that is not only used directly for Zn deposition but also functions as the current collector, thereby significantly simplifying the production processes.^[^ ^7^, ^8^ ^]^ To unify the description in this study, we use “anode‐free” to represent the “Zn‐free” anode. Compared to the other methods applied to improve the Zn utilization level, anode‐free configurations can limit the Zn corrosion reactions, due to the minimal amount of Zn that is exposed to the electrolyte.^[^ ^9^ ^]^ Indeed, in such an anode‐free battery, the charging process involves the de‐intercalation of Zn^2+^ ions from the cathode and subsequent deposition of these ions on the current collector. Since all the active Zn^2+^ ions are stored in the cathode material, and there is no oversupply of Zn^2+^ ions as in traditional zinc ion batteries, the Coulombic efficiency (CE) becomes crucial. Generally, the plating/stripping process on the current collector exhibits lower CE compared to that of an insertion cathode. Therefore, the challenge is to improve the Zn plating efficiency, which is primarily determined by the side reactions (such as the hydrogen evolution reaction), and the dendrite growth.^[^ ^10^ ^]^ Such an improvement relies on optimizing both the current collector and the electrolytes.

MXenes have attracted many research interests in AZMBs due to their high conductivity, open‐layered structure, and unique surface chemistry. MXenes (M_n+1_X_n_T*_x_*, where M represents early transition metals, X is C and/or N, and T*_x_* represents surface functional groups (─O, ─OH, ─F)) are a family of 2D transition metal carbides and/or nitrides, synthesized by selectively etching the A layer from MAX ceramic precursors.^[^ ^11^, ^12^ ^]^ Their rich surface groups make them both hydrophilic and zincophilic, lowering the energy barrier and facilitating uniform Zn deposition.^[^ ^13^, ^14^, ^15^ ^]^ Moreover, owing to its hexagonal structure inherited from the precursor MAX phase, MXene has minimal lattice mismatch with the (002) facet of Zn, which crystallizes in a hexagonally closed‐packed structure. As such, MXene can forester the deposition of thin hexagonal flakes of Zn oriented horizontally to the film surface and thus inhibit a desired morphology for achieving cycling longevity.^[^ ^16^ ^]^ Compared to other MXenes such as Ti_2_CT*_x_*, Nb_2_CT*_x_*, Nb_4_C_3_T*_x_*, and V_2_CT*_x_*, Ti_3_C_2_T*_x_* has demonstrated the highest electronic conductivity (≈24 000 S cm⁻¹) alongside great chemical stability.^[^ ^17^ ^]^ As the most extensively studied MXene, Ti_3_C_2_T*_x_* has demonstrated exceptional potential for enhancing the Zn deposition reversibility when used as a coating layer for Zn metal or substrates for Zn pre‐deposition.^[^ ^18^, ^19^, ^20^ ^]^ For instance, by pre‐electroplating 2 µm Zn on Ti_3_C_2_T*_x_* film in 2 m ZnSO_4_ electrolyte, a self‐freestanding Ti_3_C_2_T*_x_* MXene@Zn paper with better electrolyte wettability and lower overpotential than Zn metal can be obtained.^[^ ^14^ ^]^ The MXene@Zn paper enables a dendrite‐free Zn deposition and good cycling stability with a high average Coulombic efficiency (ACE) of 94.13% for Zn plating/stripping.^[^ ^14^ ^]^

In addition to serving as surface modifiers for Zn electrodes, MXene films offer significant potential as current collectors in anode‐free aqueous zinc metal batteries (AZMBs), which could greatly enhance energy density. Their lower material density, combined with a superior affinity for Zn plating compared to traditional metallic current collectors like Cu and Ti, makes self‐standing Ti_3_C_2_T*_x_* films especially advantageous. Despite this potential, there are currently no reports of Ti_3_C_2_T*_x_* films being directly employed for Zn plating/stripping in AZMBs, though they have shown notable performance as current collectors in other energy storage systems.^[^ ^21^, ^22^ ^]^ This absence may stem from challenges in achieving high Zn deposition reversibility on pure MXene films, with prior studies primarily exploring composite‐based electrodes instead. For instance, A flexible 3D Ti_3_C_2_T*_x_*/graphene aerogel fabricated by the directional freezing method has been utilized directly as the framework for Zn plating/stripping.^[^ ^23^ ^]^ The 3D porous structures are capable of effectively encapsulating Zn, leading to a dendrite‐free Zn deposition on Ti_3_C_2_T*_x_*/graphene. Consequently, superior reversibility for Zn plating and stripping with an ACE of ≈99.67% upon 600 cycles at 10 mA cm^−2^ was achieved. Even at a high deposition capacity of 5 mAh cm^−2^, Zn is uniformly deposited inside the micropores of the MXene/graphene 3D framework with minimal granular protrusions on the surface, which significantly prevents the formation of Zn dendrites. Combining Ti_3_C_2_T*_x_* with Antimony (Sb) or nanocellulose to prepare MXene‐based composite as a current collector for AZMBs also demonstrated good reversibility for Zn plating/stripping.^[^ ^24^, ^25^ ^]^

Addressing this gap could open new pathways for MXene applications in AZMBs. Rationally designing electrolyte systems could enhance the reversibility of Zn plating on Ti_3_C_2_T*_x_* self‐freestanding films in anode‐free AZMBs, offering a solution that maintains a straightforward electrode production process. Optimizing the electrolytes, by modifying the salt concentration, and introducing ionic additives, organic molecules, and polymers, stands out as another practical solution to facilitate the Zn deposition on Zn metal or other metallic current collectors.^[^ ^7^, ^26^, ^27^, ^28^ ^]^ The modification of electrolytes can change the solvation structure, influencing charge transfer and the Zn^2+^ ion transport process. This, in turn, affects the nucleation and growth of Zn on the electronic conductive substrates.^[^ ^29^, ^30^ ^]^ Additionally, some additives may participate in the formation of solid electrolyte interphase (SEI), concurrently inhibiting the hydrogen evolution reaction (HER), side reactions, and dendrite growth.^[^ ^31^, ^32^, ^33^ ^]^ Optimizing electrolytes for reversible Zn plating on Ti_3_C_2_T*_x_* presents unique challenges compared to conventional electrolyte design strategies aimed at enhancing Coulombic efficiency (CE) on metal surfaces. This challenge arises from the intercalation process of ions and subsequent deposition, both of which are dependent on the electrolyte composition. Moreover, the presence of intercalated ions can affect the charge distribution within the MXene's structure, further impacting the subsequent Zn deposition behavior. Therefore, traditional electrolyte designing strategies that promote reversible Zn deposition on metal may not work for 2D Ti_3_C_2_T*_x_* MXenes. This motivates studies toward the influence of electrolytes on the Zn deposition behavior on MXenes, which to the best of our knowledge, has yet to be explored.

**Figure 1:**
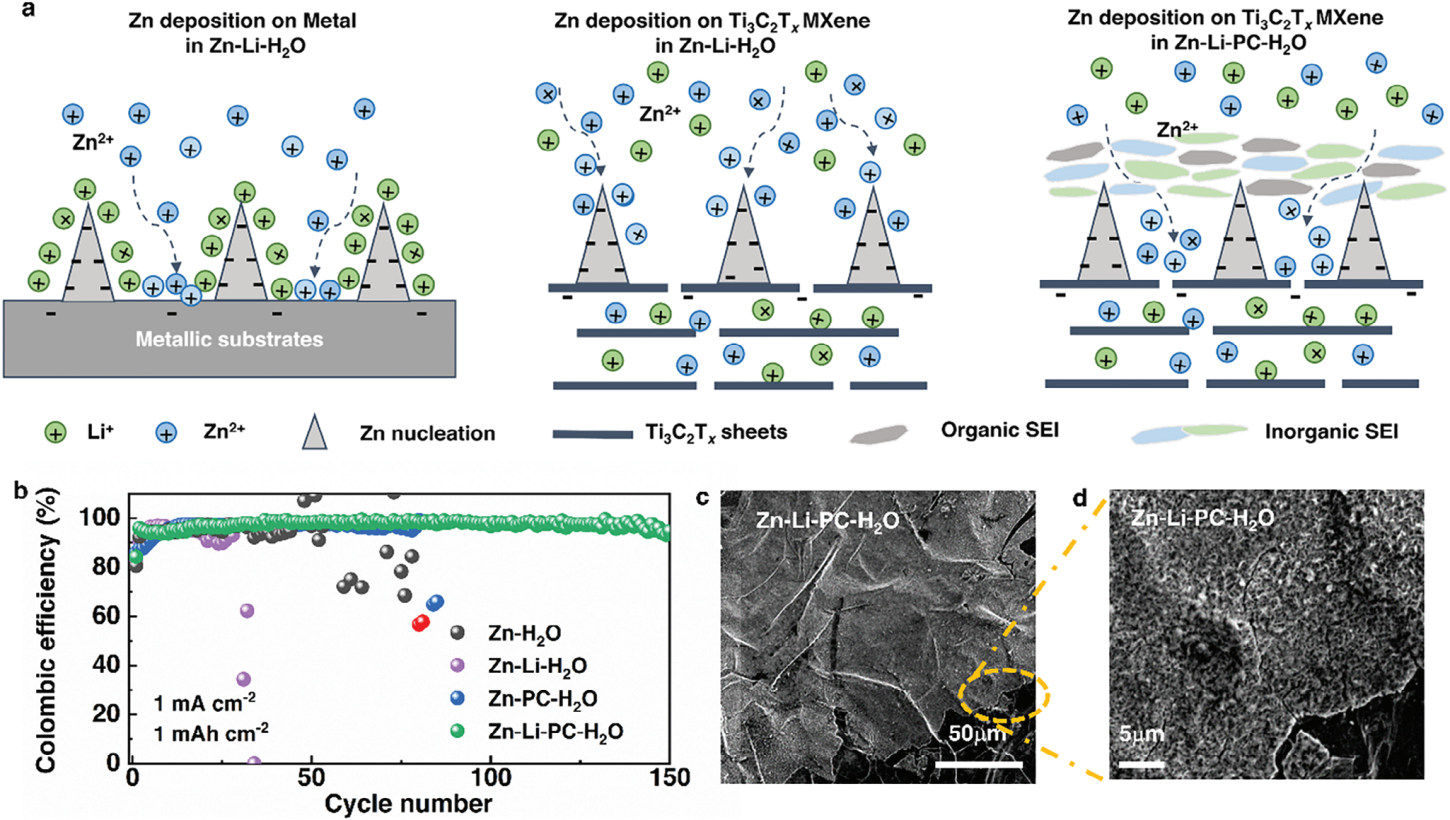
a) Schematic illustration of the Zn deposition process on Metal substrates, Ti_3_C_2_T*_x_* MXene surface in Zn‐Li‐H_2_O electrolytes, and the Zn deposition process on Ti_3_C_2_T*_x_* MXene in Zn‐Li‐PC‐H_2_O electrolytes. b) Coulombic efficiency measurements of Ti_3_C_2_T*_x_*//Zn cell with a deposition capacity of 1 mAh cm^−2^ at a current density of 1 mA cm^−2^ in different electrolytes. c,d) SEM images of Ti_3_C_2_T*_x_* electrode after plating 1 mAh cm^−2^ of Zn at 1 mA cm^−2^ in Zn‐Li‐PC‐H_2_O.

In this work, we demonstrate that the co‐addition of LiTFSI (Li‐salt) and polyethylene carbonate (PC) into Zn(OTF)_2_ electrolytes (denoted as Zn‐Li‐PC‐H_2_O) can significantly improve the reversibility of Zn plating/stripping on Ti_3_C_2_T*_x_* MXene due to the formation of ZnF_2_‐containing SEI layer. The Li‐salt is selected since Li^+^ ions are capable of homogenizing the ion flux on the metal surface due to the electrostatic shielding effect, leading to a uniform metal deposition (**Figure** **1**a left).^[^ ^34^ ^]^ PC is chosen as an aprotic organic solvent that interacts strongly with both H_2_O molecules and ions, which may affect the ion intercalation process and/or the interfacial properties.^[^ ^35^ ^]^ Using either Li‐salt or PC as additives has been reported to facilitate the reversible Zn^2+^ ion deposition on metal.^[^ ^7^, ^34^ ^]^ On 2D Ti_3_C_2_T*_x_*, surprisingly, we find that the addition of Li‐salt alone (Zn‐Li‐H_2_O) unexpectedly leads to severe dendritic Zn growth, possibly due to the intercalation of Li^+^/Zn^2+^ into 2D Ti_3_C_2_T*_x_* sheets (Figure 1a middle). When combining PC and Li‐salt as co‐additives, although the intercalation behavior of cations was not changed, we observed the formation of a ZnF_2_‐containing organic/inorganic hybrid SEI layer. This ZnF_2_‐containing SEI layer functionalizes as an effective charge regulator, leading to homogenous Zn deposition (Figure 1a right). Consequently, reversible and uniform Zn plating was realized in Zn‐Li‐PC‐H_2_O electrolytes on the Ti_3_C_2_T*_x_* surface with a high maximum CE of 97.8% and an ACE of 96.8% (including the first cycle) over 150 cycles (Figure 1b–d) in a Zn‐Ti_3_C_2_T*_x_* half‐cell. This work demonstrates that the impact of electrolytes on Zn deposition behavior is different on 2D materials than on metal surfaces. It also provides a general strategy for rational electrolyte design to improve the reversibility of Zn plating on Ti_3_C_2_T*_x_* MXene for anode‐free AZMBs.

## 2 Result and Discussion

### 2.1 Zn Deposition Behavior on Self‐Freestanding Ti_3_C_2_T*_x_*

Ti_3_C_2_T*_x_* films were obtained by selectively etching Ti_3_AlC_2_ through a mild in‐situ HF method reported earlier and a subsequent filtration process.^[^ ^36^ ^]^ The as‐resulted Ti_3_C_2_T*_x_* films were flexible and self‐freestanding (Figure S1, Supporting Information), thus they were directly employed as the current collectors for Zn plating/stripping in anode‐free AZMBs. As shown in the X‐ray diffraction pattern (Figure S2, Supporting Information), the disappearance of peaks attributed to Ti_3_AlC_2_ and the downshift of (0 0 2) peaks indicates successful synthesis of Ti_3_C_2_T*_x_*. Additionally, the Ti_3_C_2_T*_x_* films display a significantly preferred orientation along the c‐axis, which is beneficial for the Zn growth along the (0 0 2) plane.^[^ ^20^, ^24^ ^]^ The successful exfoliation was further confirmed by Raman spectroscopy and X‐ray photoelectron spectroscopy (XPS) (Figure S3, Supporting Information).

**Figure 2:**
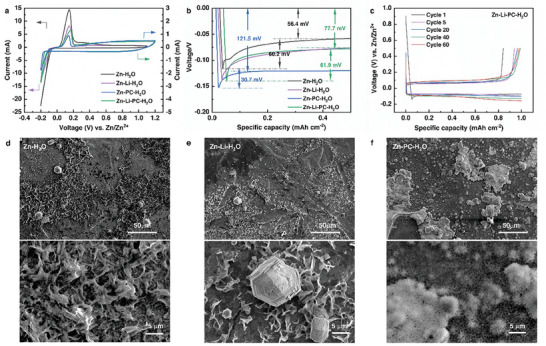
a) CV curves of Ti_3_C_2_T*_x_*//Zn cell in Zn‐H_2_O, Zn‐Li‐H_2_O, Zn‐PC‐H_2_O, and Zn‐Li‐PC‐H_2_O electrolytes at a sweep rate of 0.5 mV s^−1^. b) Voltage profiles (1st cycle) and corresponding nucleation overpotential values of Ti_3_C_2_T*_x_*//Zn cell at a current density of 1 mA cm^−2^ in different electrolytes. c) Charge/discharge curves of Ti_3_C_2_T*_x_*//Zn cell in Zn‐Li‐PC‐H_2_O (Capacity: 1 mAh cm^−2^, current density: 1 mA cm^−2^). SEM images of Ti_3_C_2_T*_x_* electrode after plating 1 mAh cm^−2^ of Zn at 1 mA cm^−2^ in d) Zn‐H_2_O electrolyte and e) Zn‐Li‐H_2_O electrolyte f) Zn‐PC‐H_2_O electrolyte.

To investigate the deposition behavior of Zn on Ti_3_C_2_T*_x_*, cyclic voltammograms (CV) were recorded using a Ti_3_C_2_T*_x_*//Zn half‐cell with a scan rate of 0.5 mV s^−1^ in 1 m Zn(OTF)_2_ electrolyte (Zn‐H_2_O) (**Figure** **2**a)*_._* A slightly larger initial Zn plating/stripping potential located at −0.08/0.15 V was observed, compared to −0.055/0.105 V in 2 m ZnSO_4_ electrolyte,^[^ ^14^ ^]^ due to the large size of OTF^−^ than SO_4_^2−^. The Zn plating/stripping reversibility was then evaluated by galvanostatic discharge/charge profiles with a deposition capacity of 1 mAh cm^−2^ (at a current density of 1 mA cm^−2^) and a stripping cutoff voltage of 0.5 V. A low initial CE of 80.0% was observed in the Zn‐H_2_O electrolyte, possibly due to HER and dendrite formation. Moreover, only moderate cycling stability was achieved, with an ACE of 94.5% and a short lifetime of just 40 cycles (Figure 1b).

To evaluate the impact of Li‐salt additive on the Zn deposition process, 1 m LiTFSI was added to the Zn‐H_2_O electrolyte to prepare a Zn‐Li‐H_2_O electrolyte (1 m LiTFSI + 1 m Zn(OTF)_2_ in water). Li‐salt additive was reasonably chosen since the Li^+^ ions are capable of enhancing the Zn deposition/stripping reversibility on Zn metal due to the electrostatic shielding effect.^[^ ^34^ ^]^ Li^+^ ion, has a more negative deposition potential than Zn^2+^ ion and is known to create a positively charged electrostatic shielding around the initial growth tip.^[^ ^37^, ^38^ ^]^ In turn, this can homogenize the Zn^2+^ ion flux on the dendrite surface (Figure 1a left) and thereby inhibit further dendrite growth on metallic substrates. Unexpectedly, the CE became more fluctuating, and the Ti_3_C_2_T*_x_*//Zn half‐cell can only be cycled 25 times with an ACE of 90.6% (Figure 1b). Additionally, the Ti_3_C_2_T*_x_* displayed a larger nucleation overpotential (61.9 mV) and deposition overpotential (77.7 mV) in Zn‐Li‐H_2_O electrolytes compared to that obtained in Zn‐H_2_O electrolytes (Figure 2b). The nucleation overpotential gradually decreased and stabilized at the 5th cycle (Figures S4 and S5, Supporting Information), attributed to better electrolyte wettability upon cycling.

The morphologies of the deposited Zn on Ti_3_C_2_T*_x_* with a deposition capacity of 1 mAh cm^−2^ were further examined using scanning electron microscopy (SEM) analysis. Zn flakes that protrude upward with large irregular Zn agglomerations were observed on the Ti_3_C_2_T*_x_* surface in both pristine Zn‐H_2_O electrolyte (Figure 2d; Figure S6, Supporting Information) and Zn‐Li‐H_2_O electrolyte (Figure 2e; Figure S7, Supporting Information). This observation suggests that the addition of Li‐salt alone does not effectively function as a charge regulator. The redox‐active Ti_3_C_2_T*_x_* with large interlayer spacing allows the intercalation of different cations, contributing to pseudocapacitance. Previous reports showed that Zn^2+^ ions can reversibly (de‐)intercalate in Ti_3_C_2_T*_x_*, leading to a Zn^2+^ ion storage capacity of 78.4 mAh g^−1^ at 0.2 A g^−1^.^[^ ^39^ ^]^ Such an intercalative behavior may lead to a different charge distribution on the Ti_3_C_2_T*_x_* surface than on regular metallic current collectors, since, in the latter case, the electric double layer is formed only on the metal surface.

The intercalative behavior of MXene was accessed by collecting CVs in Zn‐H_2_O and Zn‐Li‐H_2_O electrolytes within a narrower voltage window, where no Zn^2+^ ion deposition occurs. Figure S8 (Supporting Information) shows the CVs of Ti_3_C_2_T*_x_* in Zn‐H_2_O and Zn‐Li‐H_2_O electrolytes. Ti_3_C_2_T*_x_* displayed a pair of redox peaks at 0.75/0.83 V in the Zn‐H_2_O electrolyte. Additionally, a higher current density was observed when the cell was discharged to 0.3 V versus Zn. Correspondingly, a broad oxidation peak at 0.3 V during the charging process can be observed. The presence of peaks in CV is in good agreement with the sloping region observed in the voltage profile in Figure S4 (Supporting Information), contributing to an intercalative capacity of 0.05 mAh cm^−2^ at the 1st cycle. Therefore, the first deposition process of Zn on MXenes involves two steps: the intercalation of Zn^2+^ ions into the interlayer spacing of Ti_3_C_2_T*_x_* at a relatively positive potential and the subsequent Zn plating process on the Ti_3_C_2_T*_x_* surface at more negative potential. This intercalation process is partially irreversible, as evidenced by the significant decrease in intercalation capacity observed in the following cycles (Figure S4, Supporting Information). When Li^+^ ions are introduced into the electrolyte, a different electrochemical feature with a much broader and less intense oxidation peak at 0.35 V was observed, possibly caused by the de‐intercalation of Zn^2+^ and/or Li^+^. Also, considering the similar sizes of Li⁺ and Zn^2+^, Li⁺ ions can be easily intercalated into MXene before Zn^2+^ deposition occurs. XRD and inductively coupled plasma (ICP) analysis are conducted to examine the d‐spacing change and the Li⁺ content change after Zn deposition. The (0 0 2) diffraction peak of Ti_3_C_2_T*_x_* located at 2θ = 5.84^°^ and 5.77^°^ in Zn‐H_2_O, and Zn‐Li‐H_2_O electrolyte, corresponding to d‐spacings of 1.51 and 1.53 nm, respectively (Figure S9, Supporting Information). Although the XRD results do not provide direct insight into Li⁺ intercalation, the slight increase in d‐spacing may suggest a potential difference in intercalative behavior. Furthermore, ICP analysis indicates a reduction of Li⁺ concentration in the electrolytes after the first discharge process (Table S1, Supporting Information), supporting our assumption of Li^+^ co‐insertion into the Ti_3_C_2_T*_x_* prior to Zn metal deposition. Consequently, Li^+^ ions likely reside within the interlayer spacing of Ti_3_C_2_T*_x_* rather than accumulate on the surface, thereby fail to form an effective positive electrostatic field near the surface and limit their ability to regulate Zn^2+^ ion flux (Figure 1a). Additionally, the presence of TFSI^⁻^ anions in Zn‐Li‐H_2_O electrolytes may adversely affect Zn deposition reversibility. When TFSI^⁻^ was replaced with OTF^⁻^ at the same concentration, the CE and cycling performance of Zn plating on Ti_3_C_2_T*_x_* in Zn‐LiOTF‐H_2_O electrolyte, while not superior, were comparable to those in Zn‐H_2_O electrolyte (Figure S10, Supporting Information).

Achieving an effective regulation of Zn deposition by Li^+^ ions requires rational modification of the absorption behavior of Li^+^ ions on the MXenes surface. Such a modification can be realized by tuning the solvation structure of the electrolyte. As a commonly used solvent in Li‐ion batteries, PC shows high polarity and a high dielectric constant. Therefore, PC can interact strongly with cations and water molecules through dipolar interaction, enabling effective modification of solvation structure. As a result, introducing PC as a co‐solvent in the Zn(OTF)_2_ aqueous electrolyte has been shown to significantly improve the CE of Zn plating/stripping on Cu foil, raising it from 92.5% to 99.7%.^[^ ^7^ ^]^ Moreover, it has been shown that using PC as a solvent may induce a different intercalative behavior of Li^+^ ions into Ti_3_C_2_T*_x_*.^[^ ^35^ ^]^ Given this consideration, in our system, PC was introduced as a co‐additive into Zn‐Li‐H_2_O to prepare Zn‐Li‐PC‐H_2_O electrolyte (1 m LiTFSI +1 m Zn(OTF)_2_ in 50%PC/50%H_2_O). A more reversible plating/stripping behavior can be observed in the Zn‐Li‐PC‐H_2_O electrolyte with a high maximum CE of 97.8% and an ACE of 96.8% (including the first cycle) over 150 cycles with minimal fluctuation over 150 cycles (Figure 1b). Ti_3_C_2_T*_x_* displayed a slightly larger nucleation overpotential (61.9 mV) and deposition overpotential (77.7 mV) in Zn‐Li‐PC‐H_2_O electrolytes during the first plating/stripping (Figure 2b). One can also observe the decreased nucleation overpotential and the more negative plateau in the subsequent cycles (Figure 2c), possibly caused by dynamic differences.^[^ ^22^ ^]^ The Zn plating morphology on Ti_3_C_2_T*_x_* in Zn‐Li‐PC‐H_2_O were further investigated. A smooth and dense deposition of Zn was observed (Figure 1c,d; Figure S11, Supporting Information). When PC was used as the only additive into the electrolyte (1 m Zn(OTF)_2_ in 50%PC/50%H_2_O, denoted as Zn‐PC‐H_2_O), we observed a relatively lower ACE of 95.1%, a life span of only 80 cycles (Figure 1b), and a dendritic Zn growth behavior (Figure 2f; Figure S12, Supporting Information). Moreover, a much higher overpotential (121 mV) was observed in the Zn‐PC‐H_2_O electrolyte, due to the absence of Li‐salt.

### 2.2 Impact of Zn‐Li‐PC‐H_2_O Electrolytes on HER and Zn Corrosion

**Figure 3:**
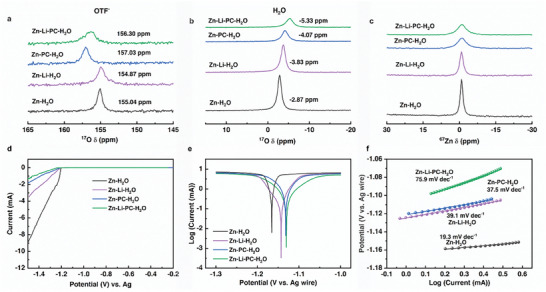
a,b) ^17^O NMR spectra for different electrolytes (Zn‐H_2_O, Zn‐Li‐H_2_O, Zn‐PC‐H_2_O, and Zn‐Li‐PC‐H_2_O). c) ^67^Zn NMR spectra for different electrolytes. d) Linear polarization curves in different electrolytes at a scan rate of 5 mV s^−1^. e) Tafel polarization plots and f) corresponding linear fitted region.

To understand the diverse Zn plating reversibility on Ti_3_C_2_T*_x_* in different electrolytes, the electrolyte structures need to be first explored. Liquid‐state nuclear magnetic resonance (NMR) spectroscopy of ^17^O and ^67^Zn was performed to investigate the solvation structure of different electrolytes. In the spectrum measured on the Zn‐H_2_O electrolyte, two ^17^O peaks located at −2.87 and 155.04 ppm were observed, corresponding to the ^17^O signal in water and OTF^−^, respectively. When Li^+^ ions were introduced into the electrolyte (Zn‐Li‐H_2_O), the ^17^O signal of OTF^−^ shifted to upfield (154.87 ppm), suggesting an enhanced shielding effect due to a weaker interaction between Zn^2+^/Li^+^ and OTF^−^ (**Figure** **3**a). In contrast, the addition of PC led to an upshift (downfield) of the ^17^O peak (OTF^−^) to 157.03 ppm and 156.30 ppm in Zn‐PC‐H_2_O, and Zn‐Li‐PC‐H_2_O, respectively. This shift suggests that the presence of PC enhances the interactions between Zn^2+^ and OTF^−^, likely due to a stronger ion‐pairing effect facilitated by the reduced dielectric constant of the solvent.^[^ ^40^ ^]^ Additionally, a substantial downshift of the ^17^O signal in H_2_O from −3.83 ppm (Zn‐Li‐H_2_O) to −5.33 ppm (Zn‐Li‐PC‐H_2_O) was observed, attributed to the reduced hydrogen bond in water due to PC‐H_2_O interaction (Figure 3b), which is conducive to alleviating HER. Furthermore, as shown in Figure 3c, the ^67^Zn signal displayed significant broadening in Zn‐PC‐H_2_O and Zn‐Li‐PC‐H_2_O electrolyte, indicating a reduced Zn^2+^ mobility due to a stronger interaction between Zn^2+^ and OTF^−^ with the introduction of PC.^[^ ^7^ ^]^

The HER and Zn corrosion processes in aqueous electrolytes determine the CE and cycling stability of ZIBs. The HER behavior in different electrolytes was investigated by linear sweep voltammetry experiments (Figure 3d). The Zn‐Li‐PC‐H_2_O electrolyte demonstrated the most negative HER onset potential (−1.23 V vs Ag) and the lowest HER current densities, which is followed by the Zn‐PC‐H_2_O, Zn‐Li‐H_2_O, and Zn‐H_2_O electrolytes, indicative of the synergistic effect of Li‐salt and PC on suppressing the HER. Figure 3e,f showed the Tafel polarization plots and the corresponding fitted linear portion in different electrolytes. Typically, a higher exchange current density and a lower Tafel slope suggest stronger Zn corrosion.^[^ ^41^ ^]^ In the Zn‐Li‐PC‐H_2_O electrolyte, the corrosion of Zn metal can be partially inhibited, as indicated by the higher Tafel slope (75.9 mV dec^−1^), compared to that in Zn‐Li‐H_2_O (39.1 mV dec^−1^), Zn‐PC‐H_2_O (37.5 mV dec^−1^), and Zn‐H_2_O (19.3 mV dec^−1^) electrolyte. This improvement in the HER and corrosion behavior in Zn‐Li‐PC‐H_2_O electrolytes is beneficial for achieving better cycling stability.

### 2.3 Function of Adding PC in Zn‐Li‐H_2_O Electrolyte and the SEI Formation

**Figure 4:**
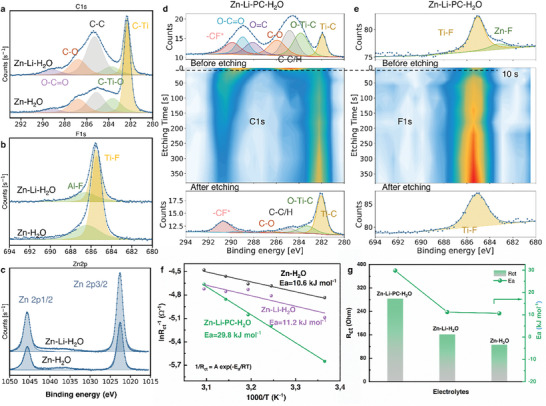
High‐resolution XPS spectra of Ti_3_C_2_T_x_ after plating 1 mAh cm^−2^ of Zn at a current density of 1 mA cm^−2^ in Zn‐H_2_O and Zn‐Li‐H_2_O electrolytes. a) C1s spectra. b) F1s spectra and c) Zn2p spectra. XPS depth profiling of Ti_3_C_2_T_x_ after Zn plating at 1 mAh cm^−2^ in the Zn‐Li‐PC‐H_2_O electrolyte. High‐resolution XPS measurements of d) C1s e) F1s of Ti_3_C_2_T*_x_*. The top panel shows a detailed scan before etching, the central panel shows a contour plot as a function of etching time where 1s of etching roughly corresponds to 0.5 nm of material. The bottom panel shows the scan after etching for 380 s. The dots indicate the measured points, and the continuous line indicates the fit to the data. f) Arrhenius plots and corresponding activation energy values for Ti_3_C_2_T*_x_*//Zn cell in Zn‐H_2_O, Zn‐Li‐H_2_O, and Zn‐Li‐PC‐H_2_O. g) The charge transfer resistance and activation energy values of the Ti_3_C_2_T*_x_*//Zn asymmetric cell at room temperature in Zn‐H_2_O, Zn‐Li‐H_2_O, and Zn‐Li‐PC‐H_2_O electrolyte.

To better understand the influence of the electrolytes on the deposition behavior of Zn on Ti_3_C_2_T*_x_*, we performed XPS to investigate the surface of MXene after Zn plating/stripping for 3 cycles in Zn‐H_2_O, Zn‐Li‐H_2_O, and Zn‐Li‐PC‐H_2_O electrolytes. **Figure** **4**a–c shows the C1s, F1s, and Zn2p spectra of Ti_3_C_2_T*_x_* in Zn‐H_2_O and Zn‐Li‐H_2_O. The C1s spectra were fitted with five peaks, corresponding to C─Ti (282.0 eV), C─Ti─O (283.3 eV), C─C/C─H (284.8 eV), C─O (286.3 eV), and O─C═O (288.7 eV) (Figure 4a), matching well with the C1s peaks of original Ti_3_C_2_T*_x_* film (Figure S3d, Supporting Information). In addition, the F1s spectra can be deconvoluted into Ti─F (685.1 eV) and Al─F (686.0 eV) signals, respectively, which are commonly observed in pure Ti_3_C_2_T*_x_* (Figure 4b). Therefore, no SEI layer was formed on Ti_3_C_2_T*_x_* after cycling in either Zn‐H_2_O or Zn‐Li‐H_2_O electrolyte. The peak observed at 1045.3 and 1022.1 eV in the XPS spectra (Figure 4c) represents the binding energies (BE) associated with the Zn2p1/2 and Zn2p3/2 orbitals, respectively, due to the intercalated Zn^2+^ into Ti_3_C_2_T*_x_*. Moreover, no significant difference in the C1s, F1s, and Zn2p spectra was observed between the surface of Ti_3_C_2_T*_x_* cycled in Zn‐H_2_O and the one cycled in Zn‐Li‐H_2_O, suggesting that the introduction of Li‐salt has a negligible impact on the interfacial chemistry.

In contrast to the Zn‐H_2_O and Zn‐Li‐H_2_O electrolytes, when PC is introduced into the Zn‐Li‐H_2_O aqueous electrolyte, a SEI layer is formed in the Zn‐Li‐PC‐H_2_O system. Introducing PC into the Zn(OTF)_2_ aqueous electrolyte as a co‐solvent can facilitate the PC and anions to enter the primary solvation sheath, which are adsorbed and decomposed on the electrode surface, and thus contribute to SEI formation.^[^ ^7^, ^42^ ^]^ In this case, the C1s spectrum of the Ti_3_C_2_T*_x_* cycled in Zn‐Li‐PC‐H_2_O electrolyte (Figure 4d) can be fitted into seven peaks, corresponding to the C─Ti (281.9 eV), C─Ti─O (283.8 eV), C─C/C─H (284.8 eV), C─O (285.9 eV), O═C (288.1 eV), O─C═O (289.0 eV) and C─F^*^ (289.9 eV), respectively.^[^ ^32^, ^43^ ^]^ The appearance of C═O and C─F^*^ clearly indicates the formation of an organic SEI layer on the MXene surface after cycling in the Zn‐Li‐PC‐H_2_O electrolyte. Additional support was found in the F1s spectrum, where an extra peak located at 683.6 eV can be observed on Ti_3_C_2_T*_x_* in Zn‐Li‐PC‐H_2_O. This peak was attributed to ZnF_2_, as the result of the reduction of OTF^−^ anions (Figure 4e).^[^ ^13^ ^]^ Furthermore, the Zn2p1/2 and Zn2p3/2 peaks located at 1044.6 and 1021.6 eV, respectively (Figure S13b, Supporting Information), showing a downshift compared to that observed in Zn‐H_2_O and Zn‐Li‐H_2_O, which may be explained by charge shift or a different interaction between Zn^2+^ and Ti_3_C_2_T*_x_* on the surface.^[^ ^20^ ^]^

XPS in combination with depth profiling using Ar^+^ etching was utilized to characterize the SEI layer. After sputtering for only 10 s, roughly corresponding to 5 nm, the intensity of C─C (C─H), C═O, and C─F^*^ of C1s signal as well as that of the Zn─F of F1s signal decreased dramatically, indicating a thin layer of organic‐inorganic hybrid SEI (Figure 4d,e). Moreover, the results of the Ti2p spectra are consistent with the formation of a thin SEI. Figure S13 (Supporting Information) shows the high‐resolution Ti2p and Zn2p XPS depth profiling of Ti_3_C_2_T*_x_* after 3 cycles in Zn‐Li‐PC‐H_2_O electrolyte. The intensity of the Ti2p peaks enhanced with increased etching time (Figure S13a, Supporting Information), which also suggests that the Ti_3_C_2_T*_x_* surface was covered by the SEI layer. An upshift of 0.7 eV in BE to 1045.3 eV (Zn2p1/2) and 1022.3 eV (Zn2p3/2) of the Zn2p spectrum was observed, as the etching time increased up to 10 s. In addition, the Zn2p signal remains after 350 s etching, indicating the intercalation of Zn^2+^ into Ti_3_C_2_T*_x_* in the Zn‐Li‐PC‐H_2_O electrolyte.

The introduction of PC may affect cation intercalation within the MXene's interlayers and influence subsequent Zn plating behavior. To investigate this, we collected XRD patterns of Ti_3_C_2_T*_x_* after Zn deposition. As shown in Figure S9 (Supporting Information), a slight reduction in d‐spacing to 1.49 nm in the Zn‐Li‐PC‐H_2_O electrolyte was observed compared to that seen in the Zn‐H_2_O and Zn‐Li‐H_2_O electrolytes. This shift may suggest that the existence of PC modified the intercalation behavior of MXene. Additionally, no obvious zinc hydroxysulfate (ZHS) byproduct was detected in all electrolytes, consistent with the idea that the anode‐free configuration effectively suppresses side reactions. Furthermore, the addition of PC to the electrolyte promoted Zn deposition preferentially on the (002) crystal plane, as evidenced by the increased intensity ratio of the (0 0 2) to (1 0 0) peaks in Zn‐PC‐H_2_O and Zn‐Li‐PC‐H_2_O, compared to that in Zn‐H_2_O and Zn‐Li‐H_2_O.

The interfacial kinetics was investigated by conducting electrochemical impedance spectroscopy (EIS) at different temperatures (Figures S14–S16, Supporting Information). Ti_3_C_2_T*_x_* showed the highest charge transfer resistance (283.8 Ω) in Zn‐Li‐PC‐H_2_O at RT (24 °C), whereas much smaller resistances were observed in Zn‐Li‐H_2_O (162.0 Ω) and Zn‐H_2_O (125.6 Ω), suggesting slower interfacial charge transfer in Zn‐Li‐PC‐H_2_O due to the SEI formation and/or the de‐solvation process. The activation energy (E_a_) values in different electrolytes are determined by the Arrhenius equation (Figure 4f):

(1) $$1/R_{ct}=A\;\exp\left(-E_{a}/RT\right)$$

where *R_ct_* denotes the interfacial charge transfer resistance, *A* represents the frequency factor, *R* is the ideal gas constant and *T* is the absolute temperature. Figure 4g displays the activation energy for different electrolytes. The activation energy is calculated to be 29.8 kJ mol^−1^ in Zn‐Li‐PC‐H_2_O electrolyte, which is almost three times as high as that in Zn‐Li‐H_2_O (11.2 kJ mol^−1^) and Zn‐H_2_O (10.6 kJ mol^−1^). The increased activation energy can be attributed to the difficult de‐solvation process when PC is present in the solvation sheath.

### 2.4 Function of Li‐Salt Additives in the Presence of SEI

**Figure 5:**
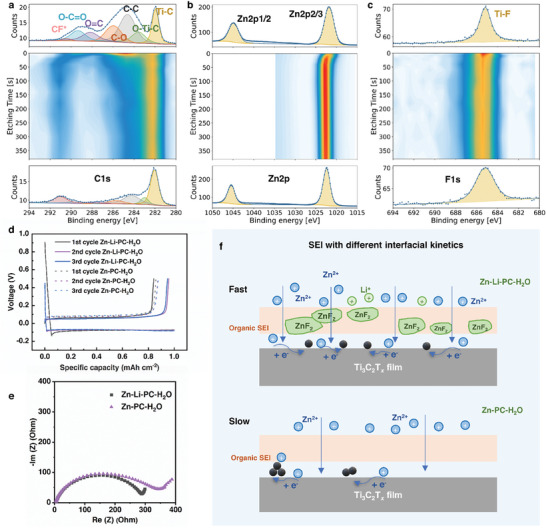
XPS depth profiling of Ti_3_C_2_T_x_ after plating 1 mAh cm^−2^ of Zn at a current density of 1 mA cm^−2^ in Zn‐PC‐H_2_O electrolyte. High‐resolution XPS a) C1s b) Zn2p c) F1s of Ti_3_C_2_T*_x_*. d) Charge/discharge curves of Ti_3_C_2_T*_x_*//Zn cell in Zn‐PC‐H_2_O and Zn‐Li‐PC‐H_2_O electrolyte of the first 3 cycles (Capacity: 1 mAh cm^−2^, current density: 1 mA cm^−2^). e) Nyquist plots of the Ti_3_C_2_T*_x_*//Zn asymmetric cell at room temperature in Zn‐PC‐H_2_O and Zn‐Li‐PC‐H_2_O electrolyte. f) Schematic illustration of the influence of diffusion interfacial kinetics in SEI on Zn deposition process on Ti_3_C_2_T*_x_* MXene in Zn‐PC‐H_2_O electrolytes and Zn‐Li‐PC‐H_2_O electrolytes.

Furthermore, the impact of Li‐salt on Zn plating behavior was investigated. In Zn‐PC‐H_2_O electrolyte, Ti_3_C_2_T*_x_* MXene exhibited higher overpotential and lower CE, compared to Zn‐Li‐PC‐H_2_O electrolyte. Additionally, dendritic Zn plating behavior was shown. **Figure** **5**d illustrates the voltage profiles of the first three cycles for Zn plating/stripping in Zn‐PC‐H_2_O and Zn‐Li‐PC‐H_2_O electrolytes. With the Zn‐Li‐PC‐H_2_O electrolyte, an initial CE of 84.3% was obtained, due to the reduction of PC and the OTF^−^ anion. The CE increased to 95.9% for the second cycle after the SEI layer was formed. In contrast, the CE in Zn‐PC‐H_2_O gradually increased from 85.8% for the initial cycle to 87.8% for the 3**^rd^** cycle, indicative of a less‐stable SEI layer, if there is any, without Li‐salt additive. Additionally, the overpotential of Zn plating significantly increased to −0.2 V after 20 cycles (Figure S17, Supporting Information), suggesting sluggish kinetics without Li‐salt additive.

The interfacial chemistry of Ti_3_C_2_T*_x_* in Zn‐PC‐H_2_O was revealed by XPS results. Figure 5a shows the high‐resolution C1s depth profiling spectra of Zn‐deposited Ti_3_C_2_T*_x_*. In the Zn‐PC‐H_2_O electrolyte, we can also observe the presence of organic SEI components (C═O and C─F^*^) due to the decomposition of PC and/or OTF^−^. Compared to Zn‐H_2_O and Zn‐Li‐H_2_O, the existence of SEI layer in Zn‐PC‐H_2_O and Zn‐Li‐PC‐H_2_O electrolyte indicates that PC is essential for the generation of organic SEI layer. Additionally, a similar upshift of binding energy can be noticed in the Zn2p spectrum with increased etching time (Figure 5b). However, no trace of ZnF_2_ was observed in Figure 5c, differing from the Zn‐Li‐PC‐H_2_O electrolyte (See Figure 4). The ZnF_2_ component is known as a superior Zn^2+^ conductor with a low Zn diffusion energy barrier of only 0.45 eV,^[^ ^44^ ^]^ which is beneficial for the homogeneous distribution of Zn^2+^.^[^ ^32^ ^]^ The existence of ZnF_2_ in the SEI layer explains the improved interfacial kinetics observed with the Zn‐Li‐PC‐H_2_O electrolyte than with Zn‐PC‐H_2_O, which is indicated by the lower interfacial charge transfer resistance observed in the Nyquist plot (Figure 5e). The Zn deposition behavior with a reduced Li‐salt concentration of 0.1 m was also examined (Figure S18, Supporting Information). This lower concentration resulted in an ACE of 91.9% over 80 cycles, along with a higher overpotential, indicating reduced stability in Zn plating/stripping and slower reaction kinetics as compared to the 1 m Li‐salt.

Therefore, the Li‐salt additive has demonstrated its significant role in the formation of a highly conductive ZnF_2_ inorganic SEI component with the co‐existence of PC, without which dendritic growth of Zn occurs (schematic illustration in Figure 5f). Based on previous results and discussions, it is reasonable to conclude that the co‐addition of PC and Li‐salt into Zn^2+^ aqueous electrolyte is essential to generate a ZnF_2_ containing organic‐inorganic hybrid SEI layer that can effectively modulate the homogenous Zn plating behavior on Ti_3_C_2_T*_x_* surface. Moreover, the as‐formed SEI layer directly regulates uniform Zn deposition, rather than redistributes the Zn^2+^ ions indirectly by influencing the intercalation behavior. Furthermore, we evaluated the Zn plating/stripping behavior on Ti_3_C_2_T*_x_* in Zn‐LiOTF‐PC‐H_2_O electrolytes, achieving an ACE of 97.0%, over 115 cycles (Figure S10, Supporting Information). This demonstrates that the type of anion has less impact on the Zn deposition reversibility in the presence of PC. Also, the presence of both Li‐salt and PC is essential for achieving high CE. In summary, the Zn deposition behavior on Ti_3_C_2_T*_x_* shows significant dependence on the electrolyte system. Specifically, the open interlayer of Ti_3_C_2_T*_x_* enables the intercalation of cations into the interlayer spacing, which may result in different surface charge distribution on MXene surface than on a metal substrate. As such, Li^+^ ions, which can effectively smooth the Zn growth on metal surfaces when serving as co‐ions of Zn^2+^, do not function properly for improving the reversibility of Zn deposition on 2D Ti_3_C_2_T*_x_* MXene. Instead, the addition of Li‐salt leads to the formation of large irregular Zn agglomerations during Zn plating. However, when Li‐salt is introduced as the additive together with PC, although the intercalation behavior is unaltered, the Li^+^ ions assist in the formation of an organic‐inorganic hybrid SEI layer. The in situ formed ZnF_2_‐containing SEI layer can effectively guide the uniform deposition of Zn (Figure 1c), resulting in a highly reversible Zn plating behavior with an ACE of 96.8% over 150 cycles (Figure 1b). When PC is introduced into the aqueous electrolyte alone, though the in‐situ formation of the SEI layer also occurs, the absence of ZnF_2_ in the SEI layer cannot effectively suppress dendritic Zn growth due to the slow interfacial charge transfer process (Figure 5f). Noteworthy, while our findings highlight the compatibility of the electrolyte system with Ti_3_C_2_T*_x_* MXene, further investigation is needed to assess the compatibility of these optimized electrolytes with other MXenes.

## 3 Conclusion

Our study demonstrates the importance of rational electrolyte design in enhancing the performance of MXene‐based anode‐free AZMBs. In our pursuit of enhancing the performance of anode‐free AZMBs, we initially explored the application of Li‐salt additive in improving Zn deposition behavior on Ti_3_C_2_T*_x_* MXene surfaces through the electrostatic shielding effect. Contrary to our expectations, this led to unstable cycling performance and the formation of irregular Zn agglomerations. However, this setback prompted further investigation into the role of electrolyte additives. Through our experiments, we discovered the critical role of Li‐salt and PC co‐additives in Zn(OTF)_2_ electrolytes. The introduction of PC reinforced Zn^2+^ coordination with OTF^−^ anion and facilitated the formation of an organic SEI layer, while Li‐salt promoted the formation of a ZnF_2_‐containing inorganic SEI layer with fast interfacial kinetics. The formation of this ZnF_2_‐containing organic/inorganic hybrid SEI layer guides a reversible and uniform Zn plating and suppresses the HER, resulting in an enhanced ACE of 96.8% for Zn plating/stripping over 150 cycles (a stabilized CE of 97.8%). Our findings highlight the differences in electrolyte design for uniform Zn plating on 2D and bulk metallic substrates and provide valuable guidelines for designing electrolytes for reversible ion plating on 2D and layered materials. A crucial future direction is to comprehensively evaluate the full cell performance of MXene‐based anode‐free AZMBs with MXene current collectors to determine how enhanced Zn deposition reversibility translates into electrochemical performance, and cycling stability for practical applications.

## 4 Experimental Section

### Materials Synthesis

The Ti_3_C_2_T*_x_* was synthesized by etching Ti_3_AlC_2_ precursor (purchased from 11 Technology Co. Ltd.) using a mixture of LiF and HCl. Briefly, 1.6 g of LiF was slowly added to 30 mL of 9 m HCl under stirring, after which 1 g of Ti_3_AlC_2_ powder was slowly added into the solution and continuously stirred at 35 °C for 24 h. The resulting solution was repeatedly centrifuged with deionized water until the pH reached ≈7. Afterward, the swollen Ti_3_C_2_T*_x_* slurry was separated from the mixture by centrifugation at 3500 rpm for 2 min. Finally, the delaminated Ti_3_C_2_T*_x_* colloidal dispersion could be obtained after ultrasonication for 1 h under the protection of Ar gas, which was followed by vacuum‐assisted filtration to get self‐freestanding Ti_3_C_2_T*_x_* film.

### Electrochemical Characterization

Ti_3_C_2_T*_x_* films were directly used as working electrodes for Zn deposition. Four different electrolytes were prepared by dissolving 1 m Zn(OTF)_2_ in water, 1 m Zn(OTF)_2_ in PC/Water (1:1 v:v), 1 m Zn(OTF)_2_ and 1 m LiTFSI in Water, and 1 m Zn(OTF)_2_ and 1 m LiTFSI in PC/Water (1:1 v:v), respectively. Electrochemical analyses were performed assembling CR2032 coin cell batteries with GF‐A film separators in Ti_3_C_2_T*_x_*//Zn half‐cell. The galvanostatic charging‐discharging processes were carried out on a Lanhe electrochemical workstation. To evaluate the Coulombic efficiency (CE) of Zn plating/stripping, a certain amount of Zn (1 mA cm^−2^ for 1 h) was deposited on the Ti_3_C_2_T*_x_* electrode and charged to 0.5 V for stripping. Cyclic voltammetry measurements were collected at a scan rate of 0.5 mV s^−1^ on a Biologic VSP‐300 potentiostat. EIS was tested at a frequency range varying from 0.001 to 100 kHz with an amplitude of 5 mV. The activation energy can be calculated from the EIS performed at different temperatures (ranging from 24 to 50 °C). Tafel plots were collected by Linear scanning voltammetry from −1.3 to −1.0 V (vs Ag) at a scan rate of 1 mV s^−1^ with a three‐electrode set‐up, where Zn foil, Carbon rod, and Ag wire was employed as reference electrode, respectively.

### Materials Characterization

Zn deposition morphology on Ti_3_C_2_T*_x_* film was studied by SEM analysis using JEOL JSM‐6010LA equipment (SED mode) at 10 kV. Additionally, energy‐dispersive X‐ray (EDX) elemental mappings were performed at an accelerating voltage of 15 kV to gain more information about the element's distribution.

X‐ray diffraction (XRD) analysis was employed to examine the interlayer space of Ti_3_C_2_T*_x_*, utilizing an X'Pert Pro diffractometer (PANalytical, operated at 45 kV and 40 mA) with Cu‐Kα radiation (λ = 1.54 Å). XRD patterns of the Ti_3_C_2_T*_x_* electrode were collected within the range of 2θ = 3–45^°^ in steps of 0.02^°^ and a speed of 6.5^°^ per minute.

NMR spectra were recorded using a Bruker 600 MHz (14.1 T) Ascend magnet equipped with a NEO console. ^1^H, ^17^O, and ^67^Zn had Larmor frequencies of 600.13, 81.36, and 37.55 MHz, respectively, at that field strength. The Zn(OTF)_2_ and LiTFSI salts were dissolved in deuterated oxide (D_2_O) for NMR measurements. 90‐degree pulse lengths of 16.2,14.7 and 25.0 µs were determined for ^1^H, ^17^O and ^67^Zn. ^1^H spectra were collected using a 30° excitation pulse followed by acquisition with a recycle delay of 2 s and 4 scans. ^17^O and ^67^Zn NMR spectra were recorded using standard single pulse experiment (Bloch decay) by collecting 1024 scans with a recycle delay time of 0.5 s and 0.2 s, respectively.

Raman spectroscopy was conducted using the Renishaw instrument with a 488 nm laser.

XPS measurements were performed with a ThermoFisher K‐Alpha spectrometer to investigate the chemical state of the elements present. The spectrometer is equipped with a focused monochromatic Al kα source (1486.6 eV) anode operating at 36 W (12 kV, 3 mA), a flood gun operating at 1 V, 100 µA, and the base pressure in the analysis chamber is approximately 2 × 10^−9^ mbar. The spot size is approximately 800 × 400 µm^2^. Etching was performed using a 3 kV Ar^+^ ion gun at a rate of ≈0.5 nm s^−1^ as calibrated on Ta_2_O_5._ The sample was etched for (cumulative) 10, 20, 40, 60, 100, 140, 180, 220, 260, 300, 340, and 380 s and the measurements were performed in “snap mode” with the pass energy of the analyzer set to 120 eV. Detailed measurements before and after etching were performed in “scan mode” with a pass energy of 50 eV. Charge referencing was performed using the hydrocarbon C1s line at 284.8 eV as the reference. All peaks were fitted using 70% Gaussian and 30% Lorentzian line shapes (weighted least‐squares fitting method) and a nonlinear Shirley‐type background using the ThermoFisher Advantage software.

Inductively coupled plasma optical emission spectroscopy (ICP‐OES) was measured using Perkin Elmer Optima 8000 DV.

## Conflict of Interest

The authors declare no conflict of interest.

## Supporting information



Supporting Information

## Data Availability

The data that support the findings of this study are available from the corresponding author upon reasonable request.
