# Supplementary material for: Enhancing Zn Deposition Reversibility on MXene Current Collectors by Forming ZnF2‐Containing Solid‐Electrolyte Interphase for Anode‐Free Zinc Metal Batteries
Source: Small. 2025 Jan 28;22(25):2407226. doi: 10.1002/smll.202407226 (PMC13137241; doi:10.1002/smll.202407226)
Supplement: Supplementary file 1 — Supporting Information [file SMLL-22-2407226-s001.docx]

Supporting Information

**Enhancing Zn Deposition Reversibility on MXene Current Collectors by Forming ZnF_2_-Containing Solid-Electrolyte Interphase for Anode-Free Zinc Metal Batteries**

Chaofan Chen^a,1^, Rui Guo^abc,1^, Swapna Ganapathy^a^, Baukje Terpstra^a^, Hao Wang^a^, Zhibin Lei^b^, Frans Ooms^a^, Bart Boshuizen^a^, Marnix Wagemaker^a*^, Lars J. Bannenberg^a*^, Xuehang Wang^a*^

^1^ Authors contributed equally to this work

^a^ Department of Radiation Science and Technology, Delft University of Technology, Delft 2629 JB, The Netherlands

^b^ School of Materials Science and Engineering, Shaanxi Normal University, 620 West Chang’an Street, Xi'an, Shaanxi, 710119, China

^c^ Xi’an Rare Metal Materials Institute Co., Ltd, Xi’an 710016, China

*Corresponding Author:

E-mail: m.wagemaker@tudelft.nl, L.J.Bannenberg@tudelft.nl>, x.wang-22@tudelft.nl


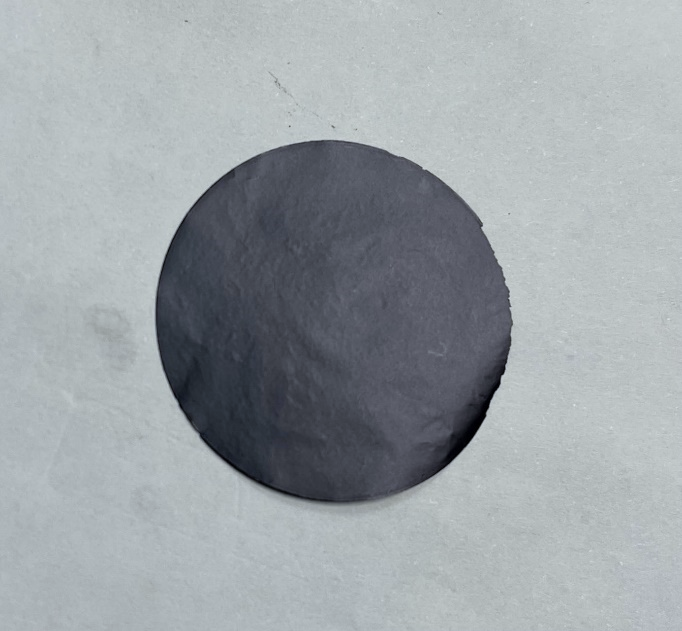


**Figure S1** Digital Photograph of the Ti_3_C_2_T*_x_* film.

**Figure S2** XRD patterns of Ti_3_AlC_2_ and Ti_3_C_2_T*_x_* film.


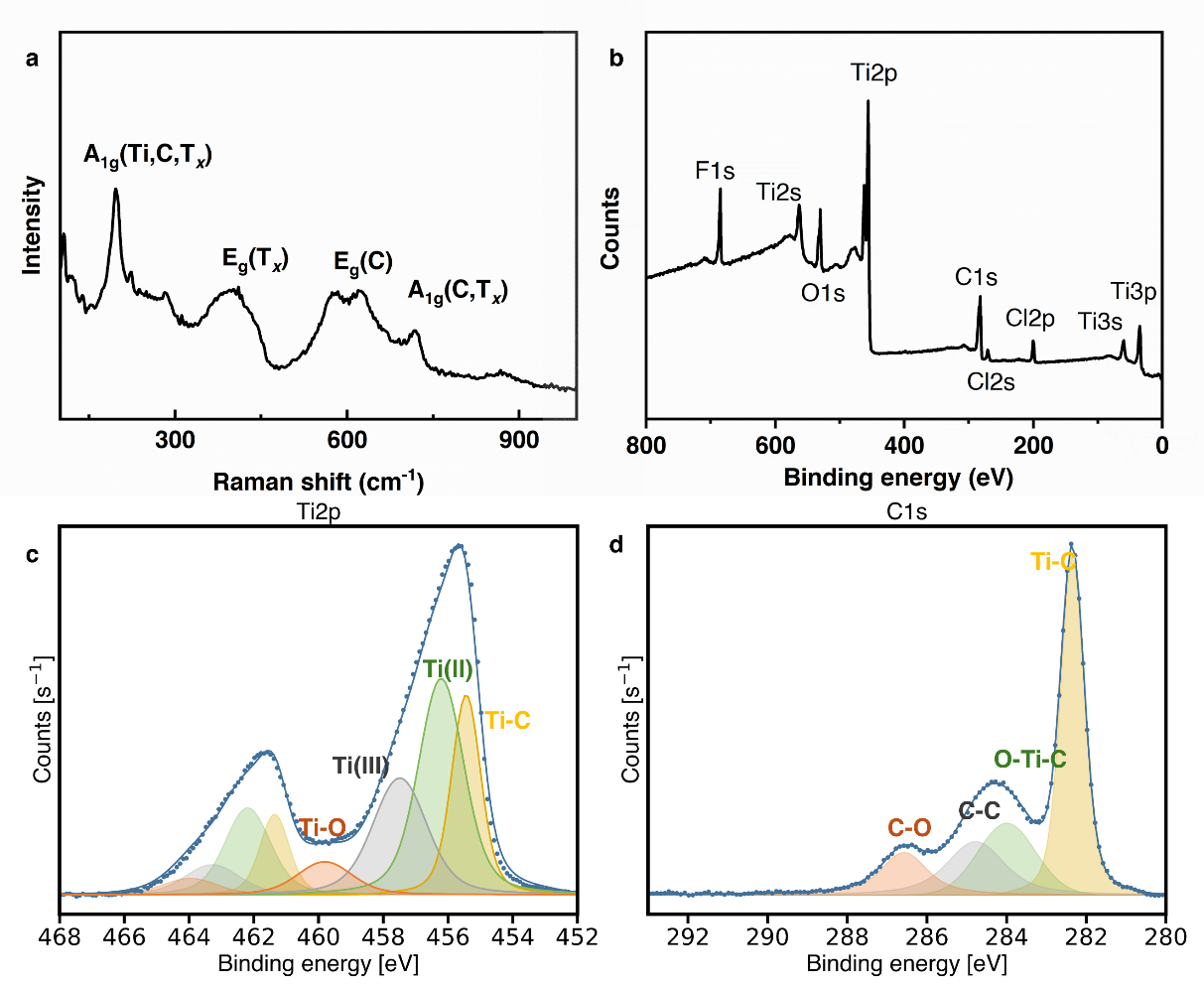


**Figure S3** Raman and XPS spectroscopy of Ti_3_C_2_T*_x_* film. (a) Raman Spectroscopy. (b) XPS Survey of Ti_3_C_2_T*_x_*. (c) High-resolution Ti2p and (d) high-resolution C1s XPS spectrum of Ti_3_C_2_T*_x_*.





**Figure S4** Selected charge/discharge curves of Ti_3_C_2_T*_x_*//Zn cell in Zn-H_2_O electrolyte (Capacity: 1 mAh cm^-2^, current density: 1 mA cm^-2^).





**Figure S5** Selected charge/discharge curves of Ti_3_C_2_T*_x_* //Zn cell in Zn-Li-H_2_O electrolyte (Capacity: 1 mAh cm^-2^, current density: 1 mA cm^-2^).


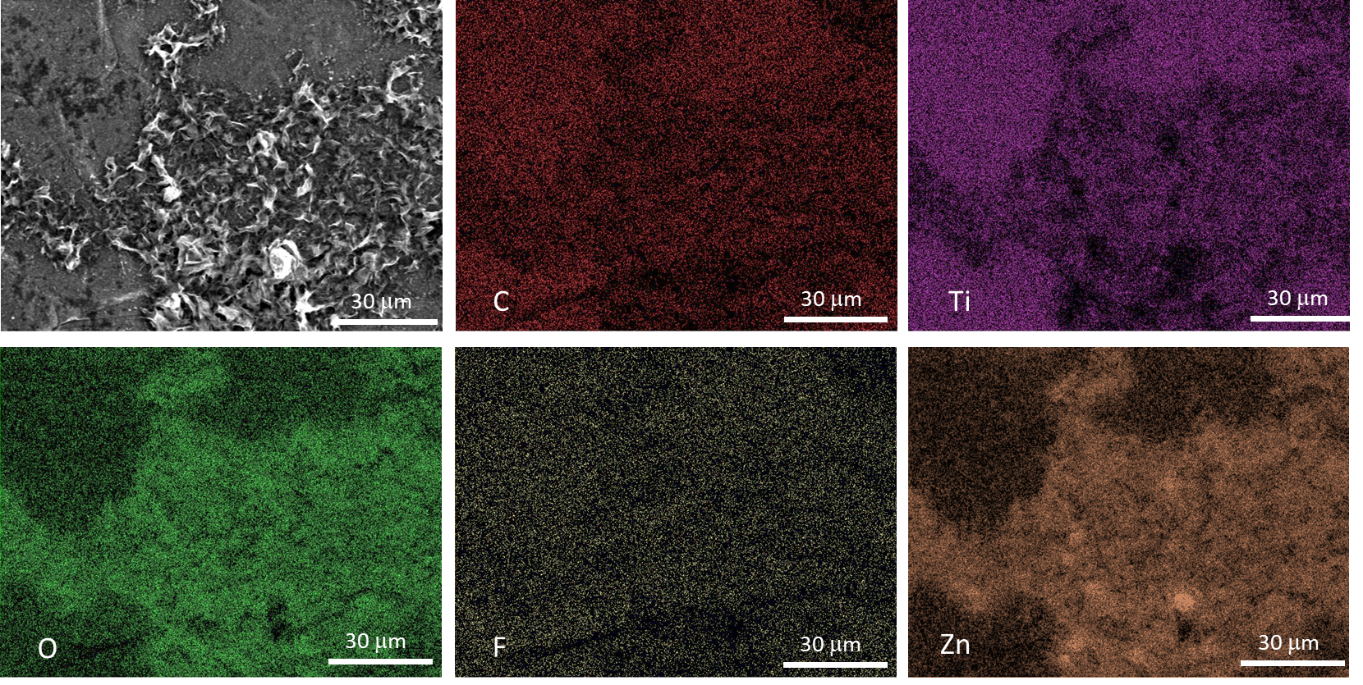


**Figure S6** SEM-EDS element mapping of Ti_3_C_2_T*_x_* after plating 1 mAh cm^-2^ of Zn at 1 mA cm^-2^ in Zn-H_2_O.


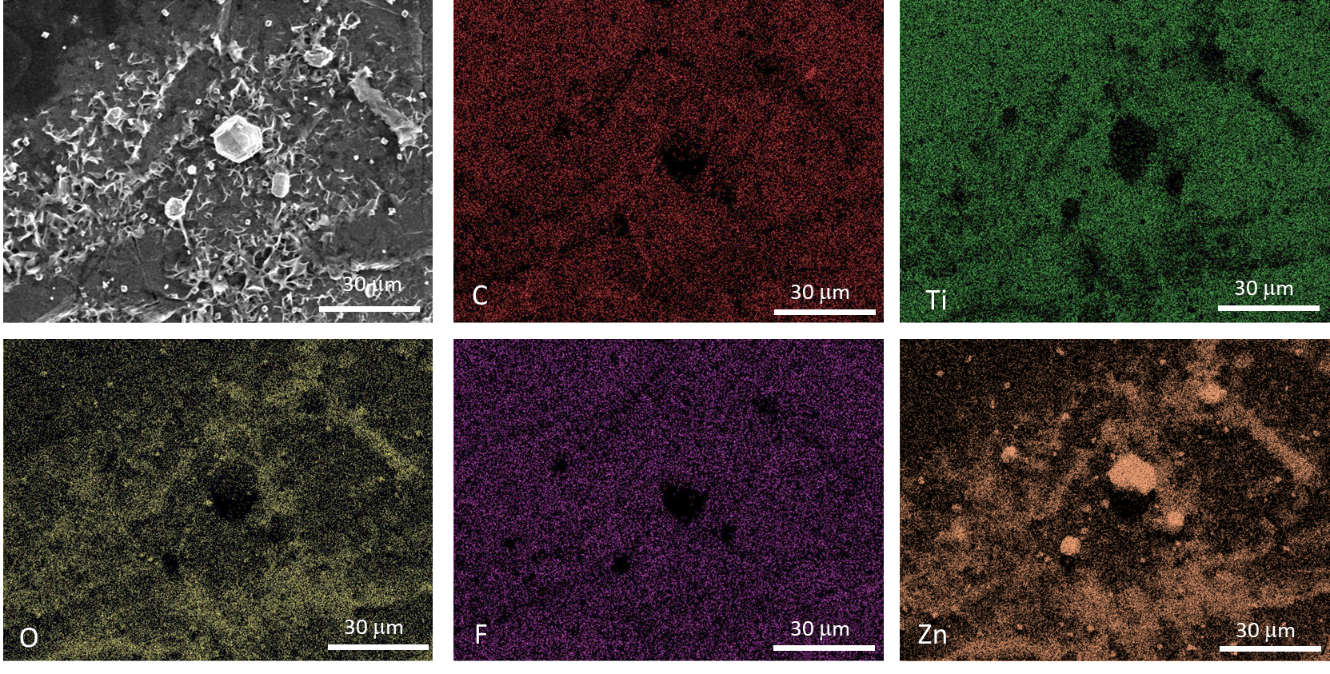


**Figure S7** SEM-EDS element mapping of Ti_3_C_2_T*_x_* after plating 1 mAh cm^-2^ of Zn at 1 mA cm^-2^ in Zn-Li-H_2_O.





**Figure S8** The CV curves of Ti_3_C_2_T*_x_*//Zn cell in Zn-H_2_O and Zn-Li-H_2_O electrolyte (3^rd^ cycle) showing the intercalative behavior at a scan rate of 0.5 mV s^-1^.

**

**

**Figure S9** The XRD patterns of Ti_3_C_2_T*_x_* deposited with 1mAh cm^-2^ Zn in different electrolytes. The symbols indicate the material to which the XRD peak corresponds to.





**Figure S10** Coulombic efficiency measurements of Ti_3_C_2_T*_x_*@Zn cell with a deposition capacity of 1 mAh cm^-2^ at a current density of 1 mA cm^-2^ in Zn-H_2_O, Zn-Li-H_2_O, Zn-LiOTF-H_2_O, Zn-LiOTF-PC-H_2_O electrolyte.


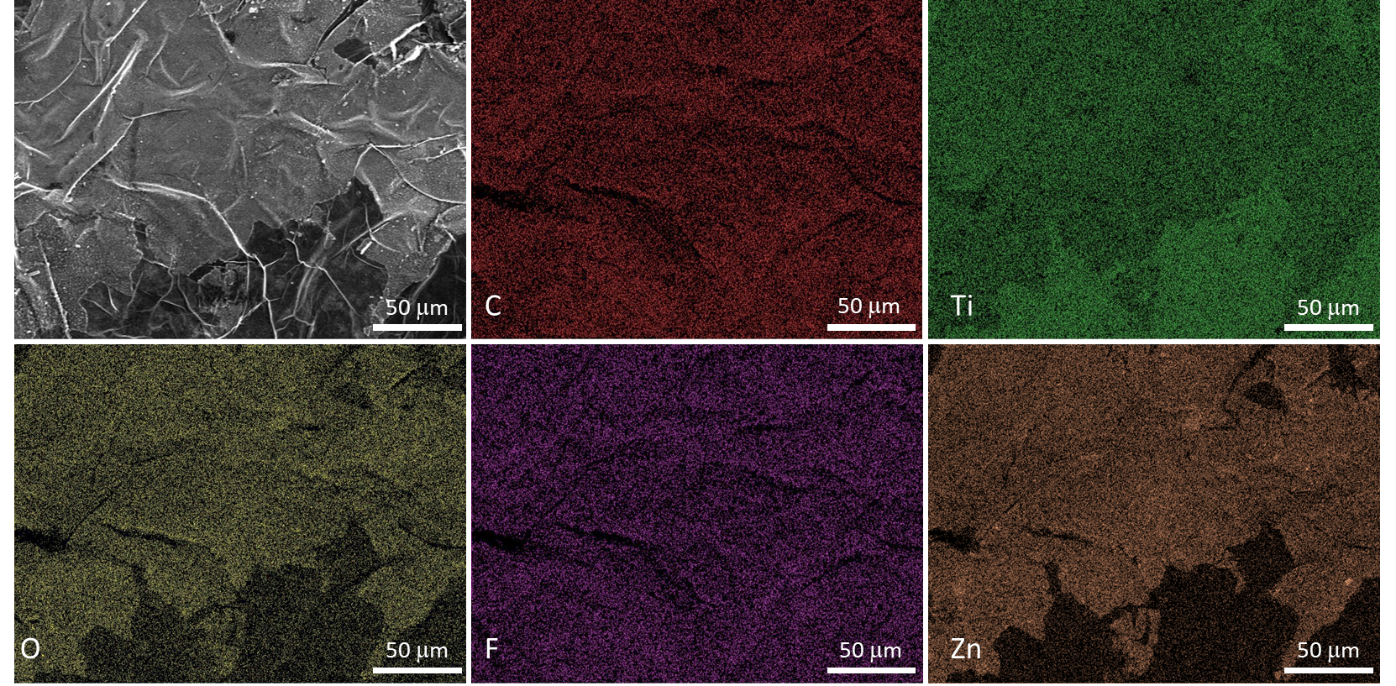


**Figure S11** SEM-EDS element mapping of Ti_3_C_2_T*_x_* after plating 1 mAh cm^-2^ of Zn at 1 mA cm^-2^ in Zn-Li-PC-H_2_O electrolyte.


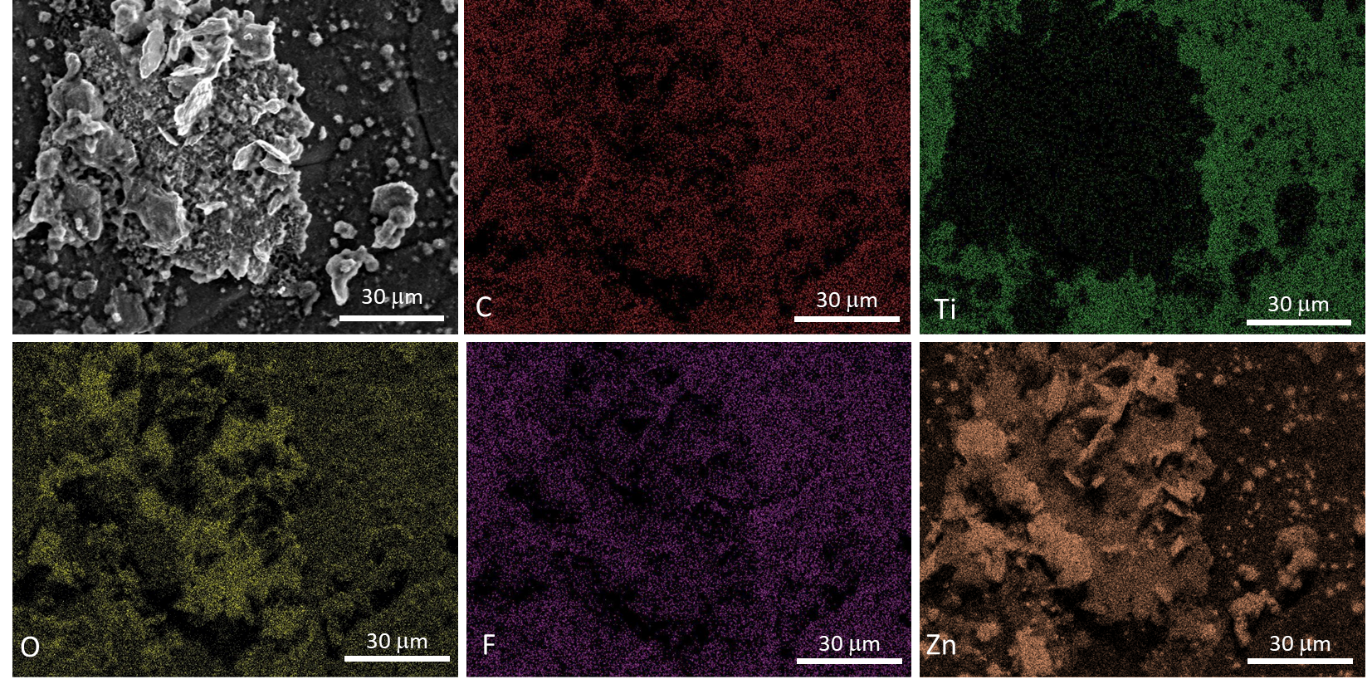


**Figure S12** SEM-EDS element mapping of Ti_3_C_2_T*_x_* after plating 1 mAh cm^-2^ of Zn at 1 mA cm^-2^ in Zn-PC-H_2_O electrolyte.


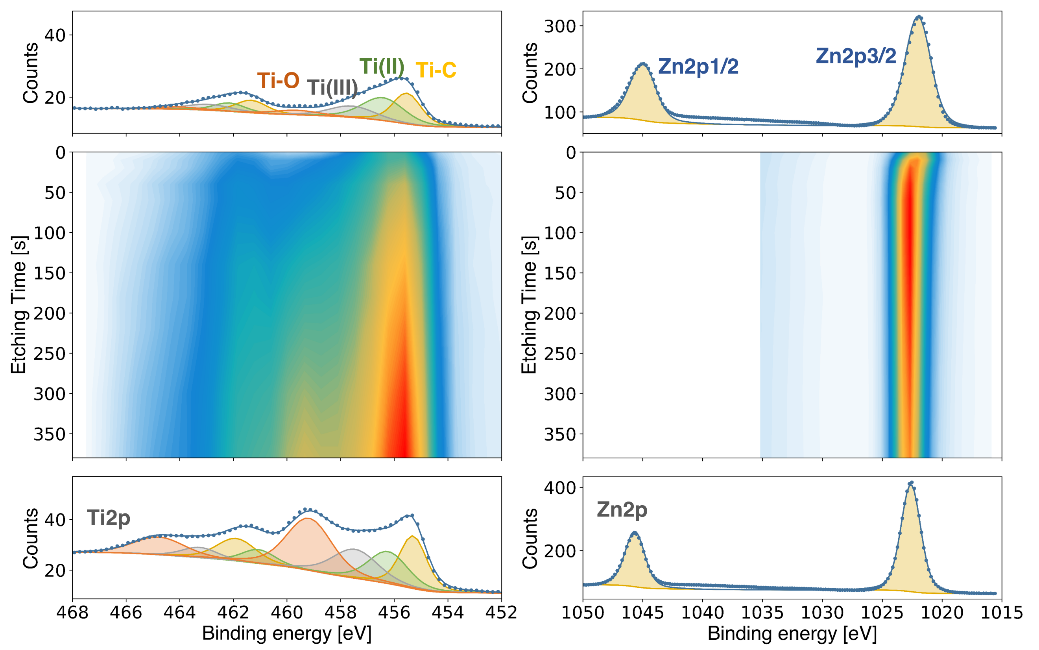


**Figure S13** (a) Ti2p and (b) Zn2p XPS depth profiling of Ti_3_C_2_T*_x_* after plating 1 mAh cm^-2^  of Zn at a current density of 1 mA cm^-2^ in Zn-Li-PC-H_2_O electrolyte.

**Figure S14** Nyquist plots of the Ti_3_C_2_T*_x_*//Zn asymmetric cell at different temperatures in Zn-H_2_O electrolyte.

.

**Figure S15** Nyquist plots of the Ti_3_C_2_T*_x_*//Zn asymmetric cell at different temperatures in Zn-Li-H_2_O electrolyte.

**Figure S16** Nyquist plots of the Ti_3_C_2_T*_x_*//Zn asymmetric cell at different temperatures in Zn-Li-PC-H_2_O electrolyte.





**Figure S17** Charge/discharge curves of Ti_3_C_2_T*_x_*//Zn cell in Zn-PC-H_2_O electrolyte of cycle 20th and 50th.


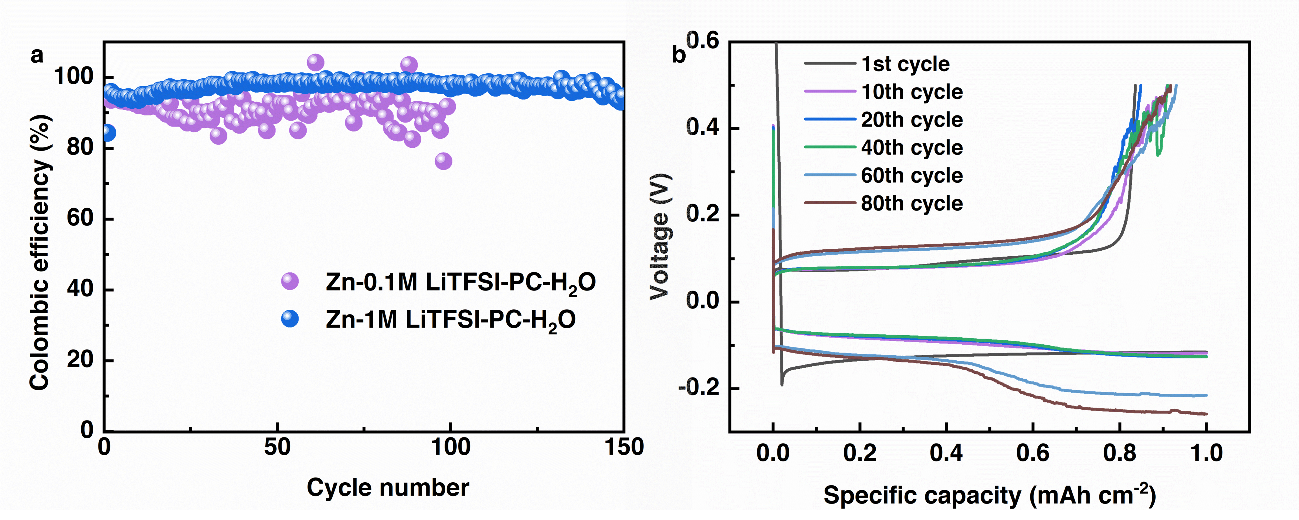


**Figure S18** (a) Coulombic efficiency measurements of Ti_3_C_2_T*_x_*//Zn cell with a deposition capacity of 1 mAh cm^-2^ at a current density of 1 mA cm^-2^ in Zn-Li-PC-H_2_O electrolyte with different concentration of LiTFSI additive. and (b) Charge/discharge curves of Ti_3_C_2_T*_x_*//Zn cell in Zn-0.1M Li-PC-H_2_O electrolyte.

**Table S1** Inductively Coupled Plasma (ICP) analysis of the Zn-Li-H_2_O electrolyte before and after the first discharge process.

| Sample (electrolyte) | Molar concentration of Li^+^ in the electrolyte |
| --- | --- |
| 1M Zn-Li-H_2_O pristine | 1.16 M |
| 1M Zn-Li-H_2_O after first discharging | 1.075 ±0.015 M |
